# A mixed-methods evaluation of capacity strengthening within an international conservation agriculture research consortium

**DOI:** 10.12688/f1000research.139715.1

**Published:** 2023-09-08

**Authors:** Kirsten Duda, Alessia D’Artibale, Miyanda Moombe, R.Murray Lark, Justin Pulford

**Affiliations:** 1 Liverpool School of Tropical Medicine, Liverpool, UK; 2 British Geological Survey, Nottingham, UK; 3 School of Agricultural Sciences, University of Zambia, Lusaka, Zambia; 4 School of Biosciences and Future Food Research Beacon, University of Nottingham, Nottingham, England, UK

**Keywords:** Conservation agriculture, capacity strengthening, consortium, evaluation, Africa

## Abstract

**Background:** The Strengthening Capacity in Environmental Physics, Hydrogeology and Statistics for conservation agriculture research (CEPHaS) consortium sought to to strengthen research capacity among a network of African and UK researchers, and their respective institutions, to fill knowledge gaps on the impacts of conservation agriculture practices on the water cycle in cultivated soils. We examined experiences of consortium membership and, drawing on this information, determined key recommendations for future programmes with similar objectives.

**Methods:** A mixed methods study encompassing an online survey (N=40) and semi-structured interviews (N=19) completed between June 2021 and February 2022 with CEPHaS consortium members from Malawi, UK, Zambia and Zimbabwe. Survey and interview data were analysed separately, using univariate statistics and framework synthesis respectively

**Results:** Survey and interview findings were generally aligned, with both revealing a wide range of reported capacity strengthening gains resulting from CEPHaS engagement at both an individual and institutional level. Participants consistently expressed their CEPHaS involvement in positive terms with praise for the applied ‘learn by doing’ approach underpinning many of the activities as well as the engaging and highly inclusive leadership. There was evidence that the various trainings and resources provided through CEPHaS were valued, frequently utilised, and often transferred beyond the immediate CEPHaS membership for wider benefit. Resource provision and staff training were seen as foundational for long-term institutional benefits. Some challenges and suggested areas for improvement were reported by participants as were potential opportunities to facilitate greater impact.

**Conclusion:** Our findings suggest that the basic ‘template’ of the CEPHaS consortium provided a strong basis for research capacity strengthening in Conservation Agriculture, especially at the level of individual researchers, and that this template could be further enhanced in any future iteration of the same or similar programme. Recommendations for replicating and enhancing CEPHaS programme strengths are presented.

## Introduction

Conservation Agriculture (CA) is a set of practices which aim to improve the sustainability of crop production and which are widely advocated in sub Saharan Africa and elsewhere as a ‘climate-smart’ strategy. ^1^ CA entails the use of zero-till, or minimum till to reduce the disturbance of soil architecture by cultivation, the use of mulches, typically crop residues, to protect the soil and enhance its organic content and biological activity, and the employment of crop rotation or intercropping to diversify the system. ^1^ Increasing global population and subsequent demand for food, combined with the pressures of climate change necessitates the adoption of sustainable agricultural practices. ^1^ With increased promotion of CA practices in Africa, both by NGOs and through government policy, there is a need to develop institutions and networks across the continent to assess its impacts on environmental systems and not just on crop yield. ^2^^,^^3^ This includes the development of interdisciplinary research capacity as an integrated assessment is needed of how CA affects soil physical properties, nutrient cycling and the water cycle. ^4^

Research capacity strengthening has been defined as “the ongoing process of empowering individuals, institutions, organisations and nations to: define and prioritise problems systematically; develop and scientifically evaluate appropriate solutions and share and apply the knowledge generated”. ^5^ As indicated by this definition, strengthening research capacity is a multi-faceted and layered endeavor that involves a comprehensive assessment and understanding of individuals and collective entities within the research environment. ^6^ In multinational research, often historic power dynamics dictate continued capacity imbalances to the detriment of sustained research capacity in low-income and middle-income countries. ^7^ To facilitate sustained research capacitation, research gaps should be identified, and the needs of all partners supported to address those gaps. ^6^

Although some research has been conducted to identify key aspects that may support multi-sectoral collaboration in CA, the same has not been done for international CA consortia. ^2^^,^^8^ There is an identified need for building stronger partnerships in CA research, but there is limited information on what is contextually beneficial or challenging for CA consortia partners. ^2^^–^^4^^,^^8^^,^^9^ Unlike the field of global health, multi-national consortia within the field of CA are less common, with research consortia primarily existing at the national level. Developing multi-disciplinary research consortia offers an opportunity for enhancing and diversifying CA knowledge and facilitating knowledge transfer across disciplines. Likewise, as food security and supporting sustainable practices have global effects, pooling knowledge internationally to develop a comprehensive and adaptive understanding of CA across geographic locations via international research consortia could provide a more nuanced and iterative understanding of CA concepts and practices.

In this paper we present findings from a mixed methods investigation of capacity strengthening experiences and outcomes as reported by members of a multi-national CA research consortium; namely, Strengthening Capacity in Environmental Physics, Hydrogeology and Statistics for conservation agriculture research (CEPHaS). The CEPHaS consortium was designed to allow for CA research to occur in parallel with capacity strengthening activities, inclusive of training and resource provision, to support individuals and institutes conducting CA in the UK and sub-Saharan Africa. We sought to identify the perceived benefits of CEPHaS participation to both research and research support staff belonging to the consortium as well as perceived benefits to their respective institutions. Challenges faced by CEPHaS consortium members were also explored with the overall aim of distilling key recommendations that may inform the implementation of similar initiatives in the future. Findings in this paper expand on a preliminary report from the same study presented elsewhere. ^10^

## Methods

### Ethical statement

Ethical approval for this study was obtained from Liverpool School of Tropical Medicine’s Research Ethics Committee (REC), UK under approval number (LSTM REC 18-038). All interview participants provided written informed consent. No incentives were provided for participation.

### Study design

A mixed methods study consisting of an online survey and semi-structured interviews (SSI), both conducted with CEPHaS consortium members from all partner countries in the final six months of the programme.

### Study setting

The CEPHaS consortium sought to strengthen research capacity among a network of African and UK researchers, and their respective institutions, to fill knowledge gaps on the impacts of CA practices on the water cycle in cultivated soils. The consortium brought together a cross-national, multidisciplinary network of researchers who worked together at sites where African partners have long-established CA trials. These included the University of Zambia’s Liempe Farm, Chitedze Research Station in Malawi and Domboshava, Zimbabwe. Through collaborative planning, installing, monitoring and data interpreting at each site, consortium members were expected to develop 1) their understanding of cross-discipline contributions to problem-solving; 2) their research skills in cutting-edge methodologies; and 3) their generic research skills (e.g., in project design or academic writing). CEPHaS delivered a wide range of training across diverse subject areas to researchers and research support staff across the CEPHaS network and supplied specialist field and laboratory equipment to consortium partners. A learning-centred structure of the demonstration studies was utilised to enable participants to take the lead in establishing similar studies or experiments both during and beyond the CEPHaS project lifetime. Further information about the CEPHaS consortium can be found at: https://www2.bgs.ac.uk/CEPHaS/.

### Sampling

The sampling frame for the survey included anyone who had been invited to attend and/or delivered a CEPHaS training between January 2018 and June 2021 and for whom an email address was held by CEPHaS project management (N=60). Survey participants were asked to indicate whether they would also be willing to participate in an SSI and, if yes, to provide their name and email address. Interview participants were purposively selected from this sample, with the objective of achieving representation across the final interview sample in terms of CEPHaS partner country, career stage, gender and position. An additional 12 CEPHaS co-investigators and staff at partner institutions, who had not been included in the survey sample, were also invited to participate in the SSIs, with the objective of ensuring representation from across CEPHaS leadership and from across a range of positions including scientific, managerial and technical. These individuals were nominated by CEPHaS project management and email addresses supplied.

### Procedure

The survey was developed by the research team and piloted with African early career researchers not belonging to the sample frame to test face and content validity of the stated questions. Minor changes to wording and survey lay out were subsequently made. The revised survey was administered via the ‘Online Surveys’ platform ( https://www.onlinesurveys.ac.uk/) and took approximately 10-15 minutes to complete. Participation was both anonymous and voluntary. Participants provided consent by selecting the ‘I consent to the survey’ option in order to access the rest of the survey. An information sheet was included with the initial survey invitation, which was sent via email with a link to the survey form. Two ‘reminder’ messages were sent, also via email. The survey remained ‘live’ online for a six-week period between June-August, 2021. Participants were able to complete the survey at any time during this period. The online survey consisted of four sections: 1) demographic and professional information; 2) investigation of uptake, utilisation and transfer of any training provided by CEPHaS as well as potential barriers and enablers to training utilisation and transfer; 3) examination of the use of any resources provided by CEPHaS as well as enablers and barriers to the use of these resources; and 4) broader exploration of participant experiences of CEPHaS participation. Survey structure consisted primarily of closed response questions with one or multiple answer options.

Prospective interviewees were invited to participate by email and, if they accepted, a suitable date and time for an interview was agreed. All SSIs were completed remotely, via a recorded Zoom call, between September 2021 and February 2022. Participation was voluntary in all cases, with each interview taking between 30-80 minutes to complete. Semi-structured interviews were informed by a topic guide which covered the same four sections as included in the survey, albeit in an open-ended format. Both the survey and interview guide were designed to elicit information pertaining to both the individual participant as well as their respective university or research institution. The two methods were considered complementary as they afforded breadth (survey) and depth (SSI) to the subsequent data analysis and allowed for data triangulation to improve reliability. All design and evaluation activities of this study were conducted by the lead author (KAD) and supported by a senior colleague (JP), based in the UK at LSTM, and both were completely independent of project activities. Survey design was reviewed by CEPHaS members (MM, AD) to inform contextual understanding and inclusion of key project areas for evaluation.

### Data analysis

Survey data were exported from online surveys into Stata/SE V.14.1 ( https://www.stata.com/) for analysis. RStudio, ( https://posit.co/products/open-source/rstudio/) a freely accessible alternative, is capable of the same analysis used in this study. Univariate analysis was performed to describe characteristics of the sample and for calculating frequencies and percentages across key focal areas of training, including knowledge/skill gain and transfer, resource use, and CEPHaS activities experienced and perceived value. All interviews were audio recorded and transcribed in full. Transcripts were entered into NVIVO 12 ( https://lumivero.com/products/nvivo/) for coding and thematically analysed using a framework synthesis approach, ^11^ informed by the interview guide. Taguette ( https://www.taguette.org/), a freely accessible alternative, is capable of the same analysis used in this study. Key themes were identified and systematically reviewed. Key themes within and across consortium dynamics were examined in a series of discussions by two investigators (KAD, JP) at key intervals within the coding cycle. Preliminary findings were presented at the CEPHaS Conference in December 2021 to help interpret the findings in the context of the programme. Survey findings have been aggregated to further protect participant anonymity. Interview excerpts presented below have been labelled with relevant participant characteristics, including profession/career stage, location and gender, to aid interpretation yet maintain anonymity. Unique participant codes, ranging from P1-P19, are also presented against interview excerpts to indicate where the same participant has been quoted more than once.

## Results

### Participant characteristics

A total of 40 respondents completed the online survey (response rate of 67%) and 19 participants completed a semi-structured interview. As shown in Table 1, survey and interview participants were predominantly male (77.5% and 78.9%, respectively), aged between 25-44 years (65% and 68%, respectively) and graduate or early career researchers (60% and 63%, respectively). Participants were relatively evenly spread between the four CEPHaS partner countries.

**Table 1.** Selected characteristics of survey (N=40) and semi-structured interview participants (N=19).

Variable	Response options	Survey	Interview
		Number (%)	Number (%)
Age	18-24	0	0 (0)
	25-34	13 (32.5)	3 (15.8)
	35-44	13 (32.5)	6 (31.6)
	45-54	10 (25)	4 (21.1)
	55+	4 (10)	2 (10.5)
	Not stated	0	4 (21.1)
Gender	Male	31 (77.5)	15 (78.9)
	Female	7 (17.5)	4 (21.1)
	Prefer not to say	2 (5)	0
Location	Malawi	10 (25)	3 (15.8)
	UK	7 (17.5)	5 (26.3)
	Zambia	12 (30)	6 (31.6)
	Zimbabwe	11 (27.5)	5 (26.3)
Position	Graduate student	7 (17.5)	1 (5.3)
	Early career researcher	17 (42.5)	6 (31.6)
	Mid-career researcher	7 (17.5)	6 (31.6)
	Senior researcher	7 (17.5)	4 (21.1)
	Research support/lab staff	2 (5)	2 (10.5)
Highest qualification	Bachelors degree	6 (15)	1 (5.3)
	Postgraduate Dip/Cert/Masters	15 (37.5)	6 (31.6)
	PhD	17 (42.5)	10 (52.6)
	Other	2 (5)	1 (5.3)
	Not stated	0	1 (5.3)

### Survey findings

Of the survey participants, 72.5% (29/40) reported attending two or more training events provided by CEPHaS, 12.5% (5/40) attended one training, and 15% (6/40) did not attend a training event (i.e., they were invited, but did not attend). The 34 participants who reported attending at least one CEPHaS training were asked to report the frequency with which they used this training and how they had applied the training. Participants who attended more than one training were asked to respond based on the training they used the most. As shown in Table 2, 50% of these 34 participants reported using their training at least monthly and 29% at least weekly. The most frequent applications of the training received were in support of the participants’ own research (68%), supporting others research (50%) or teaching within their respective institutions (38%). ‘Other’ responses included consultancy work (n=1) or application within CEPHaS (n=2).

**Table 2.** Frequency of use and type of training application (N=34).

Variable	Response options	Training	Resources
		Number (%)	Number (%)
Frequency of use	Daily	2 (6)	11 (27.5)
	Weekly	10 (29)	8 (20)
	Monthly	17 (50)	18 (45)
	Less than monthly	3 (9)	3 (7.5)
	Do not use	2 (6)	0
Training application *	Teaching within my own institute	13 (38)	19 (47.5)
	Teaching outside my institute	9 (26)	7 (17.5)
	Within my own research	23 (68)	31 (77.5)
	Supporting others’ research	17 (50)	15 (37.5)
	Community service	5 (15)	4 (10)
	Other	3 (9)	3 (7.5)

* Participants could select more than one response option.

When asked ‘apart from teaching, have you transferred this training in any other way?’, 24 out of these 34 participants responded ‘yes’. Table 3 presents the various ways in which these 24 participants reported transferring knowledge/skills gained from a CEPHaS training. As shown, knowledge/skills transfer primarily took place in the context of student supervision or supporting/supervising institutional colleagues.

**Table 3.** How CEPHaS training was transferred (N=24).

Response option *	Number (%)
Support/supervising students	17(71)
Supporting/supervising colleagues – internal	15 (62.5)
Supporting/supervising colleagues – external	9 (37.5)
Support/guiding the general public	4 (17)
Other	1 (4)

* Participants could select more than one response option.

All participants who reported attending or transferring a training were asked to identify, from a list of specified response options, enablers or barriers to utilising or transferring CEPHaS training. As shown in Table 4, the most frequently reported enablers to utilising and transferring training were ‘training was applicable to my work’, (74% and 75%, respectively), ‘having the applicable knowledge/skillset’ (62% and 71%, respectively) and ‘sufficient training’ and ‘sufficient mentorship/support’ (both 53% and 54%, respectively). The most frequently reported barriers included ‘insufficient time’ (30% and 24%, respectively) and ‘insufficient access to equipment’ (15% and 9%, respectively). However, over 50% of respondents reported ‘no barriers’ to either the use or transfer of training.

**Table 4.** Reported enablers and barriers to training application and transfer.

Response option	Training …		Resource
	Application n (%)	Transfer n (%)	Application n (%)
**Enablers**	*N=34*	*N=24* *	*N=40*
Mentorship/support	18 (53)	13 (54)	13 (32.5)
Access to equipment	7 (21)	5 (21)	21 (52.5)
Access to guidelines	12 (35)	10 (42)	20 (50)
Having the applicable knowledge/skillset	21 (62)	17 (71)	23 (57.5)
Sufficient training	18 (53)	13 (54)	21 (52.5)
Sufficient time	7 (21)	6 (25)	14 (35)
Training/resource was applicable to my work	25 (74)	18 (75)	26 (65)
Personal interest in the material/resource	19 (56)	10 (42)	21 (52.5)
Other	1 (3)	1 (4)	0
**Barriers**	*N=34*	*N=34*	*N=40*
Insufficient mentorship/support	0 (0)	1 (3)	3 (7.5)
Insufficient access to equipment	5 (15)	3 (9)	3 (7.5)
Insufficient access to guidelines	0 (0)	1 (3)	2 (5)
Insufficient knowledge/skillset	2 (6)	1 (3)	3 (7.5)
Insufficient training	2 (6)	1 (3)	4 (10)
Insufficient time	10 (30)	8 (24)	8 (20)
Training/resource not applicable to my work	1 (3)	0 (0)	0
No personal interest in the material/resource	0 (0)	1 (3)	0
There were no barriers	19 (56)	22 (65)	27 (67.5)
Other	2 (6)	0 (0)	3 (7.5)

* Only participants who reported transferring CEPHaS training were asked to report enablers.

All survey participants were presented with a list of resources supplied by CEPHaS and were asked to identify those to which: A) they had access to; B) had used; and C) had used most often (when more than one resource had been used). As shown in Table 5, most participants had access to, and had used, training materials, equipment and software supplied by CEPHaS; however, the resource used most often by 60% of participants was supplied equipment.

**Table 5.** Resource access and use (N=40).

Resource	Access n (%)	Used n (%)	Most used n (%)
Equipment	29 (72.5)	29 (72.5)	24 (60)
Software	26 (65)	26 (65)	14 (35)
Training materials	31 (77.5)	30 (75)	6 (15)
Standard operating procedures (SOPS)	21 (52.5)	17 (42.5)	1 (2.5)
No access to any of these	1 (2.5)	-	-
Other	0 (0)	1 (2.5)	0 (0)

Participants were asked how frequently they used the resources supplied by CEPHaS and for what purpose. For participants who reported using more than one resource, they were asked to respond based on the resource that they utilised the most. As shown in Table 2, almost all participants used the stated resource at least once a month or more (92.5%) most often in support of their own research (77.5%) or teaching within their own institution (47.5%). ‘Other’ responses included for practice (n=1) or were undefined (n=2).

All participants were asked to identify, from a list of specified response options, enablers or barriers to utilising CEPHaS provided resources. As shown in Table 4, the most frequently reported enablers to resource use were ‘resource was applicable to my work’ (65%), and ‘having the applicable knowledge/skillset’ (57.5%). The most frequently reported barriers included ‘insufficient time’ (20%) and ‘insufficient training’ (10%). However, 67.5% of respondents reported ‘no barriers’ to resource use.

All survey participants were presented with a list of research-related activities and asked to identify which, if any, they had experienced during CEPHaS participation; they had experienced for the first time during CEPHaS participation; and had been most useful to them and their respective institution. As shown in Table 6, ‘data analysis’ (85%) and ‘field work’ (82.5%) were the most widely reported activities, with the former also the activity most often experienced for the first time (20%) and the activity considered most useful for the individual (27.5%) and their respective institution (25%).

**Table 6.** Activities experienced during CEPHaS, experienced for the first time and most useful to the individual participant and their respective institution (N=40).

Response options	Experienced	First time *	Most – You	Most – Inst.
	n (%)	n (%)	n (%)	n (%)
Field work	33 (82.5)	3 (7.5)	9 (22.5)	7 (17.5)
Data analysis	34 (85)	8 (20)	11 (27.5)	10 (25)
Laboratory work	23 (57.5)	4 (10)	3 (7.5)	7 (17.5)
Teaching/training others	25 (62.5)	3 (7.5)	2 (5)	4 (10)
Career progression opps.	11 (27.5)	4 (10)	3 (7.5)	0 (0)
Networking/collaborations	30 (75)	7 (17.5)	14 (35)	10 (25)
Publications opps.	25 (62.5)	7 (17.5)	3 (7.5)	4 (10)
Mentorship	21 (52.5)	3 (7.5)	0 (0)	1 (2.5)
Conference presentation	9 (22.5)	3 (7.5)	1 (2.5)	2 (5)
Research support	19 (47.5)	2 (5)	3 (7.5)	5 (12.5)
Leadership responsibilities	21 (52.5)	4 (10)	2 (5)	0 (0)
Attending trainings	27 (67.5)	2 (5)	2 (5)	0 (0)
Other	0 (0)	0 (0)	0 (0)	1 (2.5)

* n=20 (50%) participants did not experience any of these for the first time.

### Interview findings

Participants described a wide range of benefits from CEPHaS membership at both an individual and institutional level. Table 7 presents a summary of these stated benefits as attributed to training provided by CEPHaS, resources provided by CEPHaS or the broader experience of CEPHaS membership.

**Table 7.** Reported benefits of consortium membership at individual and institutional levels.

Training		Resources		Experiences	
Individual	Institutional	Individual	Institutional	Individual	Institutional
Access to open source software	Facilitated partnerships	Long term access to quality research equipment & SOPs	New equipment increased efficiency of research	Broadened research perspectives	Broadened staff perspective
Developed better teaching and research practices	Research management training	Eased pressure to use other limited funds for equipment costs	Equipment expands breadth of research possibilities	Collaborative learning and research experience	Increased staff confidence
Better visual outputs	Improved interdepartmental research support	Clear guidance on equipment use & management	New equipment provided enhanced student learning opportunities	Increased confidence to communicate	Increases relevance and visibility
Exposure to new network of people	Knowledge transfer to students and colleagues	Equipment increased efficiency	New equipment adds prestige for institute	Expanded scope of personal research	Institutional assessment was conducted
Multidisciplinary training & holistic understanding	Data analysis support to other projects	Hands on experience	Reduces reliance on outside technical support	Expanded skillset, knowledge & qualifications	Institutional recognition of project quality & leadership
Hands on training	Staff equipped with field site management skills	Equipment improved quality of work	Supported interdepartmental sharing & collaboration	Provided contextual, hands-on experience	Interdepartmental transfer of knowledge & equipment
Exposure to tools outside of area of study/work	Staff trained in data quality for research outputs	Equipment expanded research possibilities	Provided well equipped lab facilities	Provided ideas for funding	Provided institutional partnerships
Facilitated a shared scientific language	Trained staff in the equipment maintenance		New equipment provides new income opportunities	Increased professionalism	Involvement in cutting edge research
Increased confidence			New equipment decreased equipment costs	Provided mentorship	Improved experiment & project management
Rare opportunity for Early Career Researchers			Partnerships created through resource sharing	Travel & cultural learning opportunities	Involved CEPHaS and non-CEPHaS staff
Provided contextual knowledge outside of subject			Created a shared data repository for research outputs	Provided international network	Exposure to international project management
Transferrable skills to other areas of research				Provided holistic understanding of CA	
Supportive learning environment				Provided a trusting and respectful environment	
				Provided opportunity for publications	
				Provided a sense of achievement and belonging	
				Observed geographical difference in equipment functionality	

Interview participants reported a range of challenges that to some extent impacted on their, or their institution’s, ability to make optimal use of (some) opportunities made available through CEPHaS membership. Challenges identified included Covid pandemic travel limitations within and between countries and the subsequent loss of face-to-face interactions; misalignment of procedures amongst institutes, and between institutes and the funders, resulting in delays in project activities; and differing partner working and communication dynamics across institutes and time-zones.

“I think I know there is always a difference between virtual meetings versus physical meetings. Because that physical relationship normally at times is important in establishing networks, that’s how I look at it … You meet, you discuss, and then you are sat somewhere on a bench, you are talking and physically you can touch this person. Yeah, it’s different from a virtual, yeah, that’s…even when you are doing all sorts of training. I think sometimes that physical one adds a bit of value than a virtual one.”

- P16, Senior Researcher, Zambia, Male

Four primary themes that positively influenced the consortium experience for consortia members emerged from the interview data, including: resource provision, strategic integration, the partnership network, and inclusivity. We discuss each theme in turn below alongside selected illustrative quotes in support of some points.

*Resource provision*

The CEPHaS project provided a multitude of resources to consortium partner institutes, including funding, equipment, software, training materials, and procedural guidelines. African institutes were the primary beneficiaries of the equipment which they could retain indefinitely. Institutes received both field and laboratory geophysics and soil science equipment that were used to rebuild and in one instance, establish a new soil science laboratory unit. The receipt of this equipment was seen as both individually and institutionally beneficial across consortium partners. Individually, members benefitted from equipment that reduced the work and time required to complete research tasks. The new equipment mechanically completed tasks that previously would have been meticulously done by hand, easing the workload of researchers and providing time to complete additional tasks.

“I used to see most of the time they had a lot of samples in the lab that needed drying then they would just remain somewhere on the benches. But now this is not the case. If you want to dry something they have bigger capacities so they can be able to do that, and much more efficiently. So, I would say generally it has really made work easier. And for most of us it has also given us that opportunity to be able to do research which maybe in most cases would be a little bit difficult.”

- P11, Mid-career researcher, Zambia, Male

Consortium members had utilized the equipment not just to conduct research for CEPHaS but research areas outside of the scope of CEPHaS, and where this had not already begun, members felt that this new equipment would enable them to expand the scope of their research and explore new areas. Likewise, having the equipment to explore new research areas was seen as a long-term benefit to the member institutes as it had the potential to expand its research portfolio. Northern consortia members, who did not receive this equipment, also saw this as beneficial to their personal research and research institutes as it meant that there were now institutes in other geographical locations that have well equipped laboratories for future collaborations and new research.

The receiving institutes were also utilizing the new equipment to support student research in individual research projects as well as existing formal courses. Consortia members at receiving institutes felt that the new equipment increased the institutes’ prestige by increasing student satisfaction, by attracting prospective students and by making them appealing to other institutes and funders as a potential research partner. Students could now have hands on experience applying the theories they learned in class, and where conducting research, students would no longer need to pay for samples to be processed at other institutions.

“Our students are also going to benefit a lot in terms of doing their practicals, to test some things in the lab … Prior to the project, our labs were not functioning well. For instance, I can mention the pressure plates. The pressure plates we use to measure the capacity of the soil to retain water were no longer functional. Thanks to the project, we managed to get those equipment that are very, very important. Especially for those students who are doing soil science and those who are doing agronomy. Because at some point, they would want to understand how the soil would be able to retain water after some amendments to it … so it’s very important for us as an institute.”

- P8, Early career researcher, Zimbabwe, Male

In some instances, this was also seen as an opportunity that could provide additional revenue streams through the provision of for-profit laboratory services which in turn would support local organizations and farmers that may benefit from these types of tests.

*Strategic integration*

The CEPHaS project was designed to capacitate institutes and individuals within the consortium and consciously incorporated complementary, integrated components into program delivery to facilitate this. Resources were provided with accompanying formal and informal training. Training started at the basics, facilitating a common scientific language across a multidisciplinary team and ensuring inclusion across demographics. Resource provision was intentionally multi-purpose. Equipment and software were provided not just to complete CEPHaS research activities, but also to establish laboratory facilities and field sites that could function as key CA research facilities in the region and provide institutes with equipment that could support revenue building services. The project framework was responsive to the context of the environment and the individuals and institutes. For example, in the initial phase of the project, it became apparent that the consortium partner institutes had different research management processes. Having aligned practices was required for the smooth transfer of funds and meeting funder requirements. The project was responsive to these needs, allocating time, resources and staff to support the training of institute staff not directly employed in CEPHaS to support project activities.

“But I think in terms of preparing the financial reports, I think people in our accounts system have benefitted. They are able to generate reports in accordance with the department or the funder. I think that experience is helpful for them when they implement other activities within the university but also even for any future or present projects in the institute.”

- P16, Senior Researcher, Zambia, Male

Staff included on the project were both permanent institute employees and employees hired specifically for CEPHaS, with the rationale to provide for the present needs of the project and capacitate sustainability beyond the end of the project life. Including permanent institute employees meant that the skills and knowledge obtained by project staff would be retained at the university and that teaching staff at teaching institutes would be able to facilitate knowledge transfer within the existing institute framework beyond the project life. Permanent institute employees also have other institute responsibilities and have time allocated across non-CEPHaS activities which could restrict availability. Employing staff outside of the institute allowed the CEPHaS project to have full time staff, while also capacitating recent institute graduates from the departments engaged in CEPHaS and other local organisations, allowing the project’s activities to support not just the consortium partners but other individuals and institutes within the local areas.

“Looking at our lab technicians, some of my colleagues where they’re academics people, they have been trained. That is a long-lasting knowledge that will be used for uplifting some of the issues that we do for our institution. For me also being trained in terms of the geophysics, it means I’m capable to say if somebody else brings in the issue of geophysics, I should be able to address and the institution will benefit or maybe they can be able to collaborate in another project.”

- P2, Senior Researcher, Malawi, Male

The project design included partners from the global north and the global south with the view to allow mutual learning across cultures and geographical context. The partners included multidisciplinary staff within the field of CA to allow for knowledge transfer across sub disciplines and allow specialists to see potential connections and develop a holistic understanding of the field. The partnerships included early-, mid- and senior career researchers. Within the project framework, staff were organized into distinct units called ‘Working Groups’ that allowed the incorporation of individuals from each of these demographics to support the inclusion of a multitude of perspectives and facilitate the creation of networks across institutes.

*Partnership network*

The partnership network created by the CEPHaS project was noted across participant members. The inclusivity, structure and duration of the project were key factors in supporting the network development. In addition to the diversity within the working groups, regular project workshops and trainings throughout the duration of the project and a range of communication mediums allowed for continuous formal and informal engagement between partners regardless of institute or geographic location. Members felt comfortable reaching out to other members through email with queries or requests for support and WhatsApp groups that allowed for both informal and formal communication. Members felt the network that was created through CEPHaS was something beneficial during the project and that it would continue beyond the life of the project, allowing for further development and research opportunities. Members felt comfortable that if queries or opportunities arose after the project, the CEPHaS members who supported them during the project would be willing to continue that engagement. These networks were seen as particularly important to early career researchers (ECRs) who could utilize this new network at a pivotal point in their career.

“And some of the contacts which I have, I think they are really important for the development of my career, which is crucial at this point as a junior researcher, I can say. I think I have long-term benefits participating in the CEPHaS … I can say some of the guys, I don’t hesitate to talk to them when I need any assistance or anything … But the network and the interaction include the whole CEPHaS team, which at times if I need anything, I can communicate with anyone without hesitation, of course.”

- P9, Early career researcher, Zimbabwe, Male

Members attributed this to trust that was developed throughout the project due to the continuous engagement between members, the length of the CEPHaS project, and the inclusivity that was generated through the project facilitation and leadership style (see below). Being a part of a project as long as CEPHaS was seen as a rare opportunity, and this allowed members to get to know working and communication styles as well as areas of expertise of other members. Additionally, although the CEPHaS project did not include PhD studentships, in at least two instances CEPHaS network membership led indirectly to scholarship opportunities for early career researchers.

“But I’d say even the scientists have learnt a lot from each other. Because as I say, the soil scientist is now appreciating the hydrogeologist, or the hydrogeologist and the shallow geophysicist are working together. They’ve instrumented a borehole and they’re both getting results from that borehole.”

- P3, Research support staff, UK, Female

Networks were facilitated across countries and supported mutual learning opportunities to develop cross-cultural professionalism and a cultural exchange of ideas to understand CA in different geographical environments.

*Inclusivity*

Inclusivity was a key feature of the CEPHaS project for consortia members. CEPHaS included both African and European institutes and individuals from a range of specialties and career levels. At the inception of the project, a workshop was held that was specifically designed to engage each member in the formation of the project plan and identify needs. Highly engaging project brainstorming activities around project deliverables were utilized. Perspectives and participation were encouraged by the project leads, facilitating a sense of value and respect amongst members regardless of position. Having this type of respect and seat at the table, from the beginning and throughout, was valuable to members and fueled their continued engagement.

“From the beginning, every member of the project was involved. We didn’t have a top-down thing. Rather, it was like, together, let’s achieve this. That was a great experience for me, how to arrange a large project of this nature, interdisciplinary or maybe cross-cultural, many countries, how to arrange it so that we could be able to work together and deliver.”

- P14, Mid-career researcher, Zambia, Female

The sense of inclusion that was generated throughout the project was attributed in part to the project leadership. The leader of the project, working groups, and the administrative staff provided verbal encouragement and support to allow members of the consortium to feel empowered, regardless of age, position or nationality. This leadership style further inspired members to want to develop a similar leadership style and incorporate elements of this approach within their own project management and teaching.

“I think it was not like a forcing, it was an encouraging. It was flexible, motivating, allowing for people to think freely, like your ideas are welcome. That was important. Even when you bring an idea, you see your idea is being debated further and sometimes even taken up. Even trainings, people were allowed, can you suggest which are the trainings you would like to attend. So, people suggest a number of trainings.”

- P14, Mid-career researcher, Zambia, Female

Another aspect that facilitated inclusivity and collaboration between members was the way members were grouped within the consortium. Small interactive working groups that reported within a defined consortia structure allowed everyone to participate. Division of tasks across demographics within working groups facilitated ongoing communication and meant all members were included and participating in the research and outputs.

This inclusive environment afforded opportunities to travel and engage in activities and discussions that were considered rare for ECRs.

## Discussion

The survey and interview findings revealed a wide range of reported capacity strengthening gains resulting from CEPHaS engagement at both an individual and institutional level. Participants consistently expressed their CEPHaS involvement in positive terms with praise for the applied ‘learn by doing’ approach underpinning many of the activities as well as the engaging and highly inclusive leadership. There was evidence that the various trainings and resources provided through CEPHaS were valued, frequently utilised, and often transferred beyond the immediate CEPHaS membership for wider benefit. Resource provision and staff training were seen as foundational for long-term institutional benefits. Some challenges and suggested areas for improvement were reported by participants as were potential opportunities to facilitate greater impact.

Perceptions highlighted in the survey and interview data were primarily aligned, with the overarching theme that the strategic integration of applicable training and resources were supportive components to participant experiences and allowed for research to be understood and conducted holistically. Provision of resources in complement with timely associated trainings and project research activities allowed for a cohesive learning experience and ensured that project activities were interconnected and consistently supported. Data analysis skills and fieldwork experiences were highly valued in both survey and interview data, with the survey data further highlighting that this was experienced by many participants for the first time. CEPHaS was viewed as a particularly valuable opportunity for ECRs to have access to hands-on multidisciplinary learning, resources, networks, and a collaborative working environment at this pivotal career point.

A primary motivating factor for participants was a supportive environment. Leadership and programme structure that facilitated inclusion were two of the key components noted by participants that fuelled personal engagement and inspiration. The inclusion of participant perspectives from the outset by the project leads, regardless of participant position, engendered respect and trust. Providing an environment and a forum in which all partners could shape the research priorities provided equity that is often lacking in North-South research environments. ^7^^,^^12^^,^^13^ Regardless of programme type, leaders setting an example of inclusion supports engagement and empowerment of its members, inspires and motivates. ^14^^,^^15^ Likewise, aligning partner interests with the goals of a project from the outset are more likely to allow for the success of a project and supports a positive learning culture. ^15^^–^^18^

Networking and collaboration opportunities were considered one of the most useful aspects of CEPHaS participation at both individual and institutional levels and participant responses suggested a high demand for additional networking opportunities over and above what was already provided. Bringing together specialists from within CA across geographies and demographics to form a multi-disciplinary team was a key benefit to CEPHaS participants. However, the initial need to train non-CEPHaS research management staff, the absence of structured knowledge transfer into institute teaching curriculum and potential for institutionally led incorporation of CEPHaS resources for revenue building activities, signify the potential value of a broader multi-disciplinary team that included additional research management and regulatory staff. Structured investment in research management staff within institutes and project structure can further allow for cohesive integration and sustained project benefits. ^15^^,^^19^ Although CEPHaS had the flexibility in project structure to allow for unforeseen financial management training, further inclusion of a strategically focussed research management working group and additional research management training may have provided insight into how potential revenue streams could have been further advanced during the project lifespan. Greater cohesion of research management functionality and staff across institutes may also have further advanced additional future funding opportunities. Structured investment in research management staff would also provide the opportunity to bridge and formalise benefits from the individuals and departments involved in CEPHaS to the wider institution.

Insufficient time was noted as a barrier within both the survey and interviews, as the quantity of information to be absorbed and utilised within the project was seen as a challenge. A recent study exploring the experiences of African postdoctoral fellows belonging to a vector borne disease research capacity strengthening consortium also identified the need to allow for additional time when a programme has both research and capacity strengthening objectives. ^20^ The lack of face-to-face interactions, primarily caused by the Covid-19 pandemic, was predominantly seen as a limitation by participants. Although virtual engagement had the benefit of recordings and scheduling flexibility, face-to-face interactions were seen as valuable opportunities to gain deeper connections with partners. The reported value of face-to-face interaction, in terms of both learning and networking, is important to consider given the recent growth and normalisation of virtual meeting and teaching delivery. ^21^

Although CEPHaS was focussed on capacity strengthening in CA research, key benefits identified by participants are consistent with benefits reported from research capacity strengthening programmes in other fields. A supportive environment, networking opportunities, and a strategically integrated approach, inclusive of methodological skills and resource provision, is important to capacity strengthening programmes regardless of topic area. ^14^^,^^16^^,^^22^^–^^24^ A supportive environment with trust and respect and inclusive decision making facilitates successful partner interactions, particularly in North-South partnerships. ^14^^,^^16^^,^^18^^,^^22^^–^^24^ Additionally, having mutual interest in the subject matter supports consortia success. ^22^^,^^24^ Although there are commonalities in capacity strengthening enablers across disciplines, many of these were reported as new and enlightening experiences within CEPHaS that shifted perspectives. As global consortia in conservation agriculture are less common than health-focused global research capacity strengthening consortia (from which much of the aforementioned evidence was drawn), our findings corroborate widely reported experiences within a new context.

Whilst a primary focus of CEPHaS was to enhance CA research capacity within the network of Southern institutes, the CEPHaS consortium was a mutually beneficial experience for both Northern and Southern institutes, evolving from intentions of capacity strengthening to actualised capacity sharing. In aspects of professionalism, networking, scientific research, and training, participants felt they benefitted regardless of geographic location. Frequently, North-South collaborations/consortia are heavily weighted to provide monetary and educational benefits to Southern institutes with Northern institutes directing activities. ^7^ Although resources were provided to southern institutes, and the training was provided to facilitate the use of these resources, partners from both the UK and African institutions were active participants in the application of this training and use of the equipment in the field setting, providing new insights into geographical differences. Northern partners gained valuable knowledge of the dynamics of their field and equipment beyond geographic boundaries, providing new areas of investigation. Mutual training and benefits to career regardless of institute are beneficial to research partnerships. ^16^ Likewise, cross-disciplinary and cross-cultural knowledge exchange was mutually beneficial in broadening perspectives and challenging operational assumptions. A consortium and research project as large as CEPHaS was seen as a rare opportunity for both institutes and individuals across locations, and for many, it was their first time being involved in a project of this size. Northern partners reported internal institutional and cross-departmental recognition of the project as a model for international consortia. Both Northern and Southern participants felt that their institutional staff had gained skills that would support future grants and project operations. The CEPHaS consortium was inclusive of elements that enabled a North-South knowledge exchange that was mutually beneficial, professionally and scientifically, and could provide a template/model supportive of capacity sharing and increased equity within global consortia, divergent from helicopter research. ^7^^,^^16^^,^^18^

The study was not without limitations, including the small sample populations (survey N=40; interviews N=19) across four countries and five research institutes. However, the mixed-methods study design did allow for some triangulation of data, allowing for greater depth and breadth than either quantitative or qualitative method would have allowed on its own and aiding confidence in the reported findings. Additionally, the study was conducted while participants were still employed by the study, and therefore, their shared perceptions may have been influenced by their ongoing engagement with the project. The study was also limited to a single CA consortium.

## Conclusion

Our findings suggest that the basic ‘template’ of the CEPHaS partnership provided a strong basis for research capacity strengthening in Conservation Agriculture, especially at the level of individual researchers, and that this template could be further enhanced in any future iteration of the same or similar programme. Key recommendations include:

• Provide inclusive leadership practices that facilitate capacity sharing;
• Strategically integrate training and resource provision within research project structure;
• Strengthen networking and collaboration opportunities, specifically for ECRs;
• Integrate research management training to develop sustainable operating structures internally and across institutes;
• Include multi-disciplinary and multi-cultural partnerships within consortia;
• Provide means for formalised inclusion of institutional level benefits to support sustainability, e.g., curricula development or commercial service provision.

## Author contributions

Conceptualisation: Justin Pulford, R. Murray Lark

Formal analysis: Kirsten Duda, Justin Pulford

Funding acquisition: Justin Pulford, R. Murray Lark

Investigation: Kirsten Duda

Methodology: Justin Pulford, Kirsten Duda, Alessia D’Artibale, Miyanda Moombe

Project administration: Kirsten Duda, Justin Pulford, Alessia D’Artibale

Supervision: Justin Pulford

Writing – original draft: Kirsten Duda, Justin Pulford

Writing – review & editing: Kirsten Duda, Justin Pulford, R. Murray Lark, Alessia D’Artibale, Miyanda Moombe

## Data Availability

Interview transcripts have not been made available as a data set because they cannot be readily de-identified without compromising anonymity. Requests for access to the interview transcripts, along with a statement as to the intended use, can be made to the corresponding author and will be considered on a case-by-case basis. Harvard Dataverse: Capacity Research, Centre for, 2023, “Data, instruments and checklists in support of publication titled: A mixed methods evaluation of capacity strengthening within an international conservation agriculture research consortium”, https://doi.org/10.7910/DVN/ANCIWR. ^25^ The project contains the following underlying data:
• Online survey dataset.xlsx. (Anonymised responses to online survey). Harvard Dataverse:Capacity Research, Centre for, 2023, “Data, instruments and checklists in support of publication titled: A mixed methods evaluation of capacity strengthening within an international conservation agriculture research consortium”, https://doi.org/10.7910/DVN/ANCIWR. ^25^ This project contains the following extended data:
• Survey instrument.pdf (Blank copy of the online survey tool). • Interview guide.pdf (Blank copy of the interview guide). Harvard Dataverse: SRQR checklist for ‘A mixed methods evaluation of capacity strengthening within an international conservation agriculture research consortium’, https://doi.org/10.7910/DVN/ANCIWR. ^25^ Data are available under the terms of the Creative Commons Zero “No rights reserved” data waiver (CC0 1.0 Public domain dedication).
