# Supplementary material for: A mixed-methods evaluation of capacity strengthening within an international conservation agriculture research consortium
Source: F1000Res. 2023 Sep 8;12:1119. [Version 1] doi: 10.12688/f1000research.139715.1 (PMC10660302; doi:10.12688/f1000research.139715.1)
Supplement: Peer review history

# Reviewer report: Reviewer response for version 1

**DOI:** 10.5256/f1000research.153014.r216406

**Published:** 2023-11-20

**Authors:** Ashim Datta

**Affiliations:** 1 WK Kellogg Biological Station, Michigan State University, East Lansing, Michigan, USA

Excellent article on capacity development and its strengthening approaches under conservation agriculture research consortia. This article provides evidence of the importance of these kind of consortia between global north and south. There are few minor corrections: 

- In abstract under "Background" section in 3rd line "to" is repeated. It should be "sought to strengthen..."
- In Introduction, first paragraph 4th line, it can be written as "enhance its organic carbon content and biological activity...."

Rest is fine. I have a few suggestions:

- The interview replies of the participants may be avoided. There are many.
- The conclusion part may be revised instead of writing bullet points with way forward and implications.

Is the work clearly and accurately presented and does it cite the current literature?

Yes

If applicable, is the statistical analysis and its interpretation appropriate?

Yes

Are all the source data underlying the results available to ensure full reproducibility?

Yes

Is the study design appropriate and is the work technically sound?

Yes

Are the conclusions drawn adequately supported by the results?

Yes

Are sufficient details of methods and analysis provided to allow replication by others?

Yes

Reviewer Expertise:

Soil carbon sequestration, GHGs dynamics, salt affected soils managment, conservation agriculture and bioenergy crops

I confirm that I have read this submission and believe that I have an appropriate level of expertise to confirm that it is of an acceptable scientific standard.

# Reviewer report: Reviewer response for version 1

**DOI:** 10.5256/f1000research.153014.r207166

**Published:** 2023-09-26

**Authors:** Bruce Currie-Alder

**Affiliations:** 1 International Development Research Centre, Ontario, Canada

A very clear article that adds to the evidence on capacity strengthening efforts. The insights on capacity 'sharing' and how both southern and northern participants benefited are particularly welcome. A few minor addition could potentially enhance the article's usefulness for those involved in designing such efforts: 

1. Please provide more detail on the content (subjects, skills, competencies) covered by the training provided. This would provide context priori to delving into survey results.
2. Provide an estimate on the size of effort under the consortium, for example, the overall financial value involved, total number/variety of training, and number of participants (while the article mentions 40+19 participants in the evaluation, this reviewer did not readily see the total number of people would participated in training). 
3. Consider reordering the rows on tables 4, 5 and 6 to present the entries from most-to-least frequently cited.
4. Consider elaborating upon the bullet list of conclusions. As written these do not yet provide a level of detail for future programme designers. An additional sentence or two for each conclusion could describe advice on what to do in future efforts and 'how to' act on these insights.  

Two additional references to consider in situating the article are Chadwick et al (2022) 'Research capacity strengthening lessons from UK funded research UKCDR, and http://hdl.handle.net/10625/58680.

 Finally, Table 7 is hard to digest as drafted, and could be presented with training/resource/experience on the vertical axis and individual/institutional on the horizontal.

Is the work clearly and accurately presented and does it cite the current literature?

Yes

If applicable, is the statistical analysis and its interpretation appropriate?

Yes

Are all the source data underlying the results available to ensure full reproducibility?

Partly

Is the study design appropriate and is the work technically sound?

Yes

Are the conclusions drawn adequately supported by the results?

Yes

Are sufficient details of methods and analysis provided to allow replication by others?

Yes

Reviewer Expertise:

Climate adaptation and resilience, research-for-development, science policy, international development, research management

I confirm that I have read this submission and believe that I have an appropriate level of expertise to confirm that it is of an acceptable scientific standard.
